# Supplementary material for: Genetic and genomic architecture in eight strains of the laboratory opossum Monodelphis domestica
Source: G3 (Bethesda). 2021 Nov 9;12(1):jkab389. doi: 10.1093/g3journal/jkab389 (PMC8728031; doi:10.1093/g3journal/jkab389)
Supplement: jkab389_Supplementary_Figures_Tables [file jkab389_supplementary_figures_tables.pdf]

Figure S1. Histograms of proportions of homozygous/ heterozygous SNPs for eight autosomes of 70 individuals in eight opossum strains.

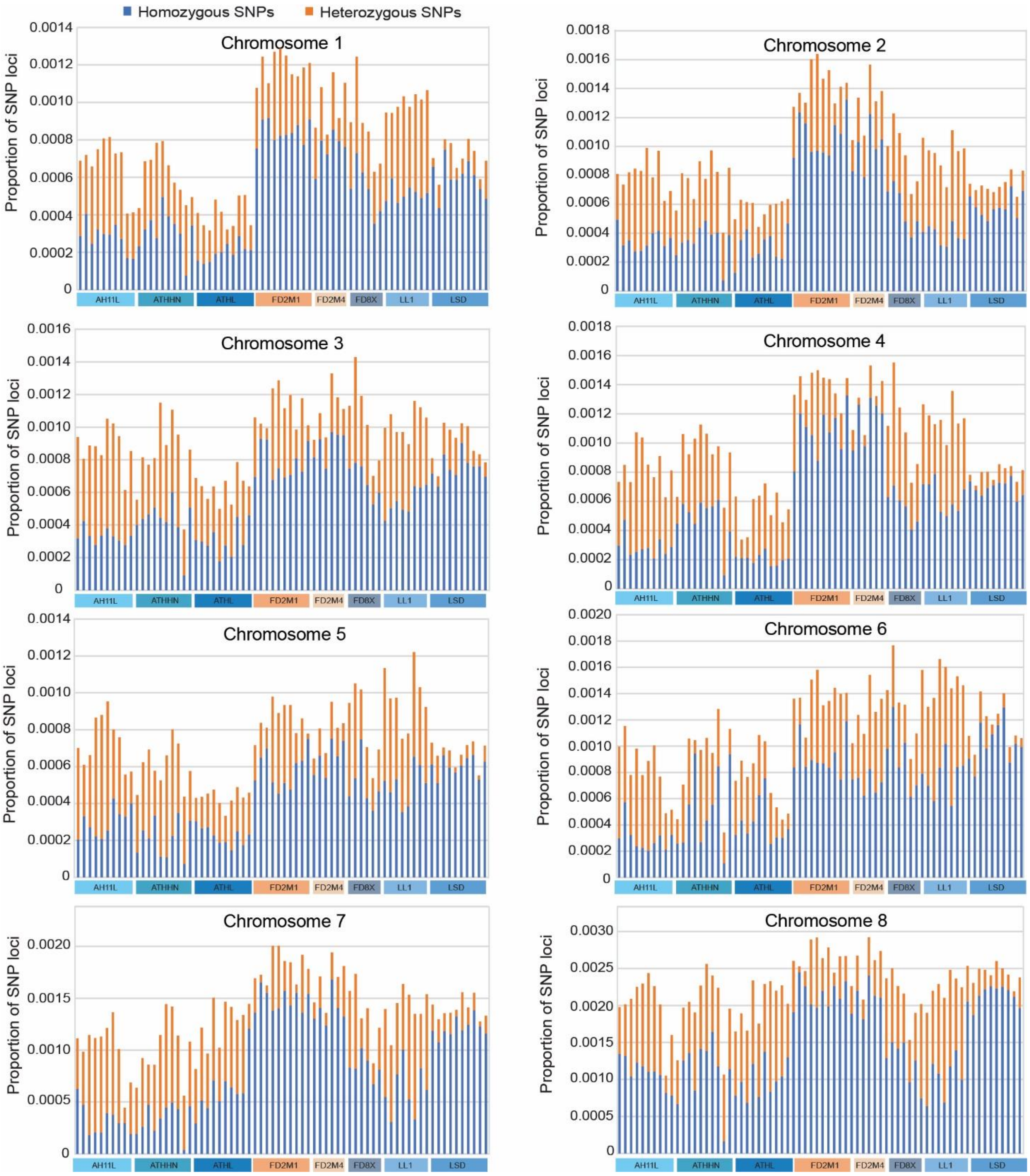

Figure S2. Barplot of numbers of heterozygous SNPs in female (*left*) and male (*right*) lab opossum samples.

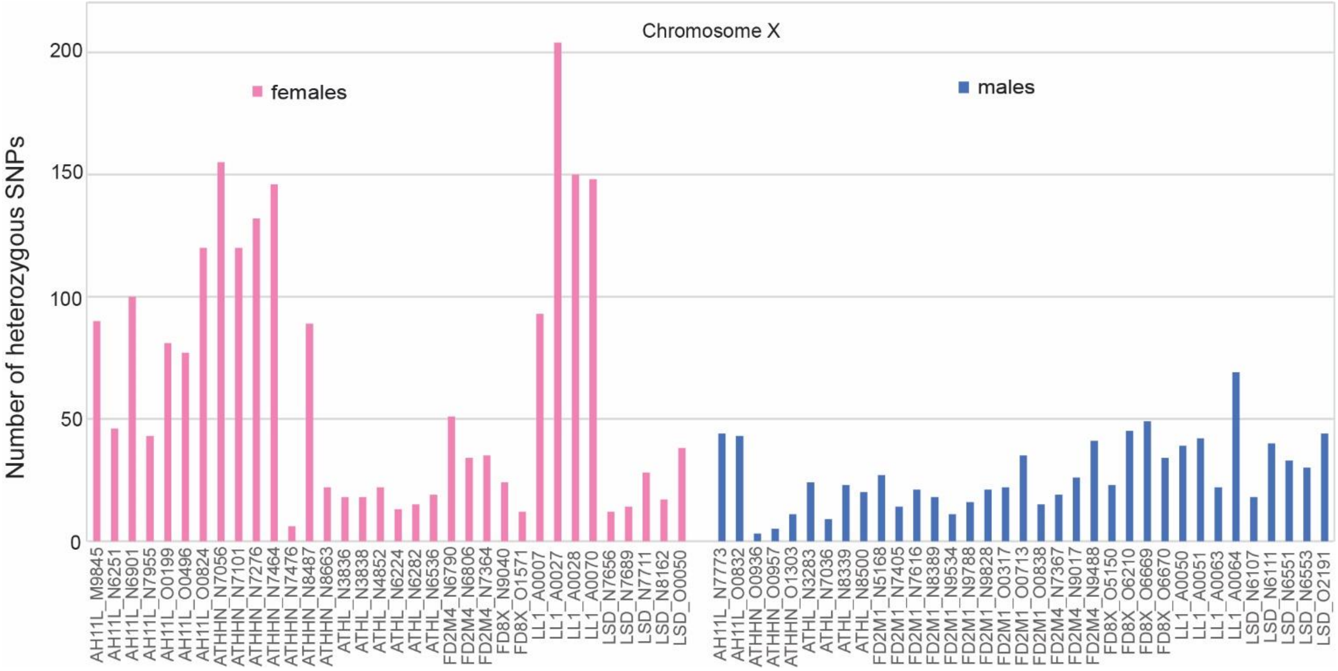

Figure S3. Window-based nucleotide diversity plots along autosomes and the X chromosome for eight lab opossum strains (window size = 20kb).

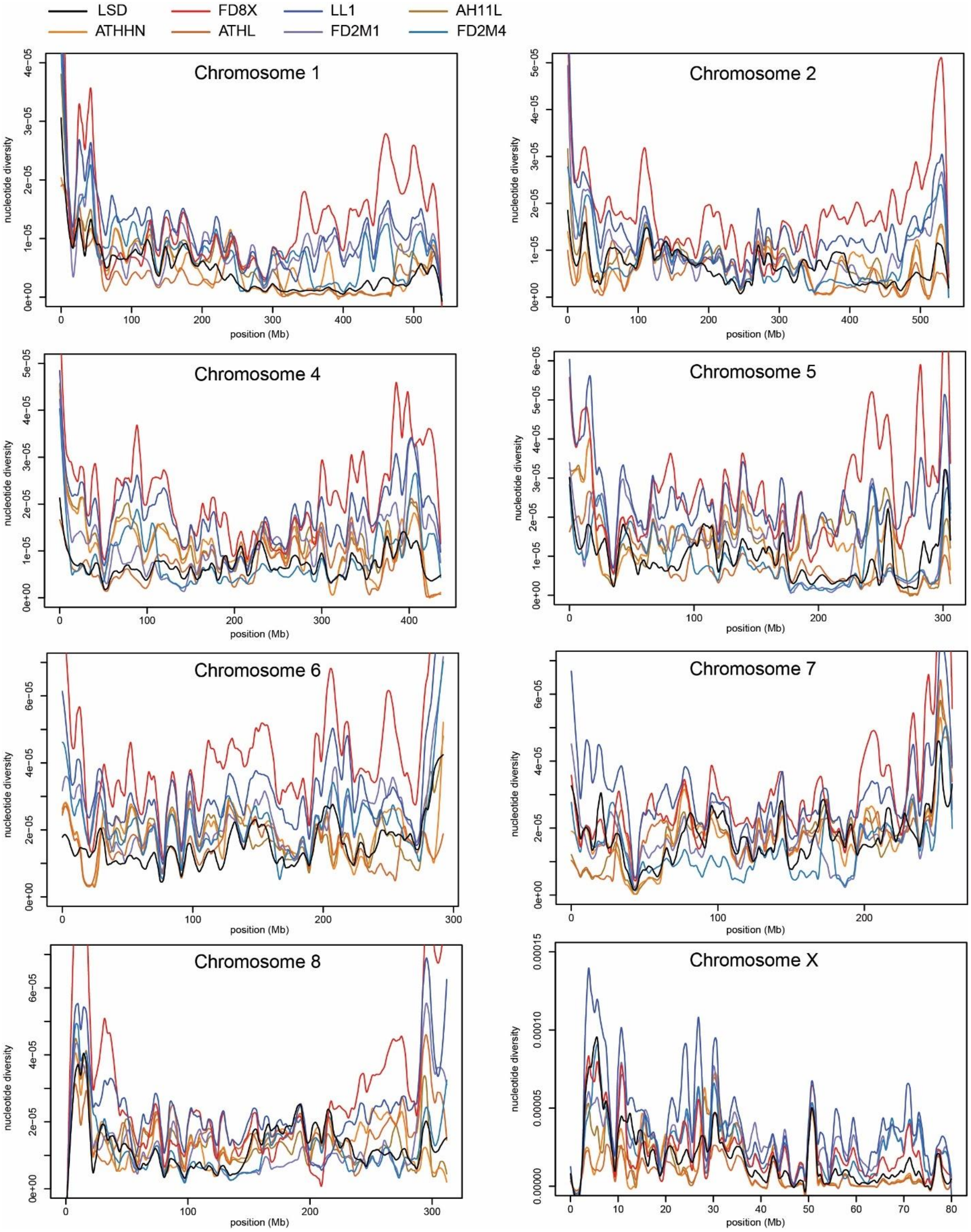

Figure S4. Window-based Fst plots along autosomes and the X chromosome for eight lab opossum strains (window size = 20kb).

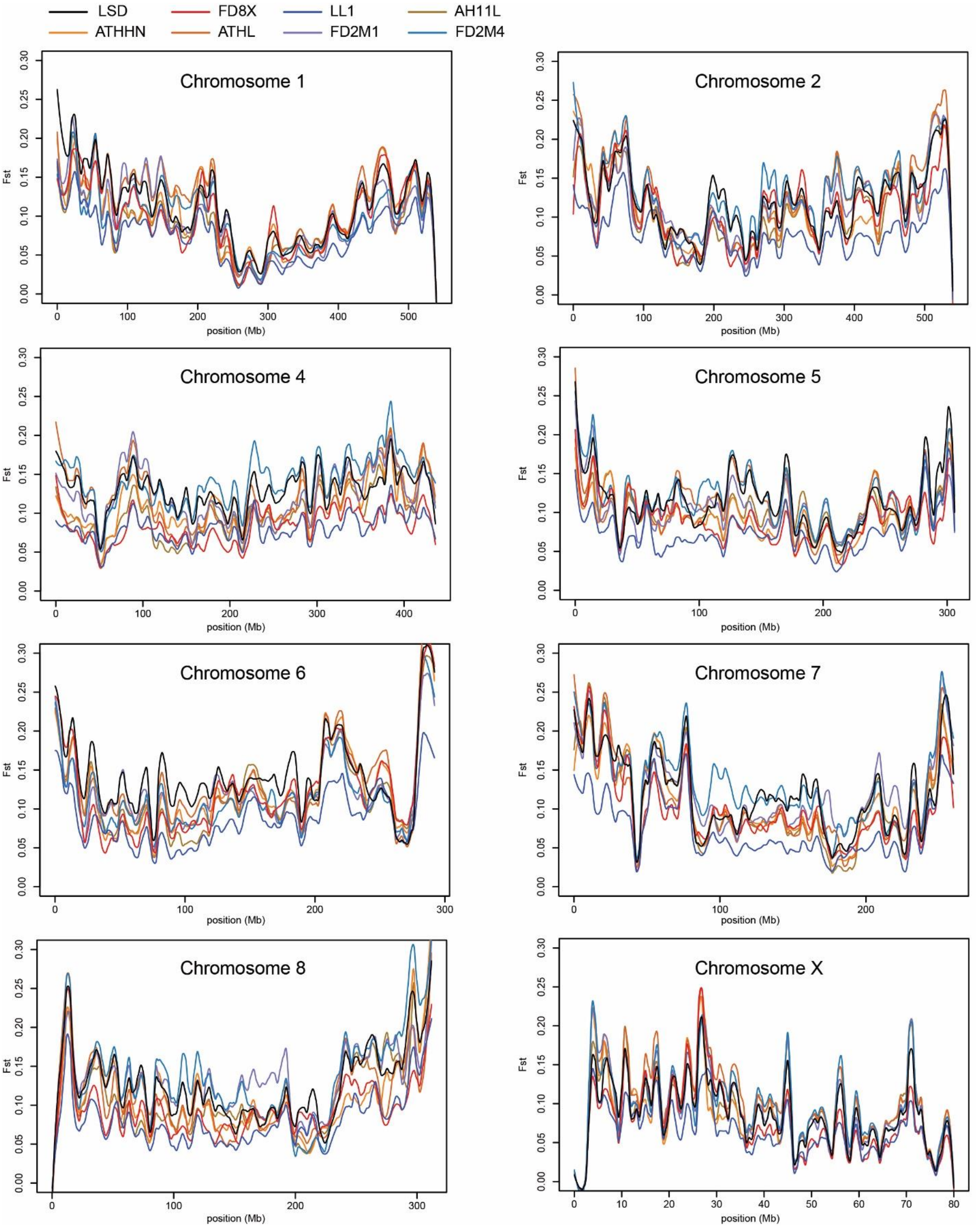

Table S1. Summary of raw read counts, quality control statistics, and genome mapping percentages for 78 ddRAD-seq individuals.

Strain	Sample ID	Number of reads	Number of reads passing QC	QC percentage	Number of mapped reads	Mapping percentage
AH11L	M9845	4,527,638	4,466,098	98.64%	4,118,600	92.22%
AH11L	N6251	8,586,782	8,416,414	98.02%	7,531,361	89.48%
AH11L	N6901	2,320,190	2,293,726	98.86%	2,117,454	92.32%
AH11L	N7773	3,358,758	3,300,518	98.27%	3,027,895	91.74%
AH11L	N7955	2,732,554	2,698,539	98.76%	2,478,313	91.84%
AH11L	N9649	2,464,380	2,426,599	98.47%	2,236,913	92.18%
AH11L	O0199	3,337,112	3,289,230	98.57%	3,035,542	92.29%
AH11L	O0496	4,360,692	4,300,758	98.63%	3,962,479	92.13%
AH11L	O0824	19,914,750	19,552,607	98.18%	17,348,618	88.73%
AH11L	O0832	14,103,210	13,950,502	98.92%	12,378,498	88.73%
ATHHN	N7056	23,013,944	22,785,140	99.01%	20,074,289	88.10%
ATHHN	N7101	4,108,190	4,059,758	98.82%	3,733,660	91.97%
ATHHN	N7276	5,313,154	5,214,857	98.15%	4,722,008	90.55%
ATHHN	N7464	9,383,856	9,287,869	98.98%	8,359,082	90.00%
ATHHN	N7476	819,502	807,207	98.50%	740,660	91.76%
ATHHN	N8487	2,302,128	2,216,219	96.27%	1,944,059	87.72%
ATHHN	N8663	991,144	970,232	97.89%	891,037	91.84%
ATHHN	O0936	1,083,236	1,069,830	98.76%	975,845	91.21%
ATHHN	O0957	1,375,396	1,344,657	97.77%	1,222,359	90.90%
ATHHN	O1303	3,201,978	3,168,500	98.95%	2,898,078	91.47%
ATHL	N3283	9,200,360	9,104,364	98.96%	8,173,174	89.77%
ATHL	N3836	8,913,378	8,820,330	98.96%	7,942,056	90.04%
ATHL	N3838	7,706,504	7,624,404	98.93%	6,964,105	91.34%
ATHL	N4852	3,796,162	3,761,234	99.08%	3,487,150	92.71%
ATHL	N6224	1,880,080	1,860,016	98.93%	1,723,735	92.67%
ATHL	N6282	3,150,848	3,104,427	98.53%	2,854,054	91.93%
ATHL	N6536	7,621,424	7,552,968	99.10%	6,838,134	90.54%
ATHL	N7036	2,788,614	2,743,543	98.38%	2,525,543	92.05%
ATHL	N8339	6,587,194	6,487,054	98.48%	5,845,618	90.11%
ATHL	N8500	7,084,630	6,999,771	98.80%	6,363,522	90.91%
FD2M1	N5168	8,662,606	8,574,896	98.99%	7,839,114	91.42%
FD2M1	N7405	5,518,832	5,465,847	99.04%	4,990,224	91.30%
FD2M1	N7616	10,004,694	9,886,088	98.81%	8,975,230	90.79%
FD2M1	N8389	3,650,256	3,618,271	99.12%	3,305,697	91.36%
FD2M1	N9534	2,739,744	2,707,836	98.84%	2,491,106	92.00%
FD2M1	N9788	6,225,322	6,140,622	98.64%	5,569,123	90.69%
FD2M1	N9828	6,020,334	5,940,445	98.67%	5,446,138	91.68%
FD2M1	O0317	10,162,876	10,064,064	99.03%	9,229,146	91.70%
FD2M1	O0713	6,743,112	6,665,832	98.85%	6,071,766	91.09%
FD2M1	O0838	3,803,700	3,713,973	97.64%	3,369,926	90.74%

Strain	Sample ID	Number of reads	Number of reads passing QC	QC percentage	Number of mapped reads	Mapping percentage
FD2M4	N6790	15,731,080	15,598,953	99.16%	13,690,280	87.76%
FD2M4	N6806	9,532,332	9,405,429	98.67%	8,570,967	91.13%
FD2M4	N7364	19,599,854	19,408,559	99.02%	17,289,754	89.08%
FD2M4	N7367	2,499,626	2,460,451	98.43%	2,253,677	91.60%
FD2M4	N9017	8,390,004	8,268,055	98.55%	7,451,272	90.12%
FD2M4	N9488	6,595,230	6,513,763	98.76%	5,956,659	91.45%
FD8X	N9040	7,656,846	7,593,079	99.17%	6,906,850	90.96%
FD8X	N9043	2,734	2,345	85.77%	1,503	64.09%
FD8X	N9044	1,202,840	1,183,637	98.40%	1,070,127	90.41%
FD8X	O0449	804,952	778,299	96.69%	697,389	89.60%
FD8X	O0452	7,614	6,347	83.36%	4,091	64.46%
FD8X	O1571	1,321,694	1,304,372	98.69%	1,181,439	90.58%
FD8X	O1762	12,282	9,934	80.88%	5,821	58.60%
FD8X	O1966	9,520	7,985	83.88%	5,136	64.32%
FD8X	O5150	3,433,830	3,402,892	99.10%	3,083,714	90.62%
FD8X	O6210	10,110,456	9,982,450	98.73%	8,773,828	87.89%
FD8X	O6669	17,739,484	17,518,371	98.75%	14,680,213	83.80%
FD8X	O6670	16,320,978	16,160,501	99.02%	14,020,571	86.76%
LL1	A0007	4,034,714	3,964,021	98.25%	3,641,522	91.86%
LL1	A0022	1,019,370	994,672	97.58%	877,980	88.27%
LL1	A0027	6,996,452	6,914,722	98.83%	6,225,990	90.04%
LL1	A0028	6,031,450	5,966,813	98.93%	5,488,543	91.98%
LL1	A0050	4,354,364	4,289,827	98.52%	3,891,697	90.72%
LL1	A0051	8,830,004	8,740,726	98.99%	8,006,584	91.60%
LL1	A0063	2,653,314	2,619,343	98.72%	2,409,122	91.97%
LL1	A0064	6,077,740	5,924,245	97.47%	5,280,979	89.14%
LL1	A0070	4,131,850	4,067,761	98.45%	3,701,978	91.01%
LL1	A0072	1,513,872	1,493,453	98.65%	1,366,910	91.53%
LSD	N6107	3,602,066	3,539,859	98.27%	3,240,569	91.55%
LSD	N6111	9,340,582	9,218,999	98.70%	8,312,296	90.16%
LSD	N6551	2,733,028	2,680,948	98.09%	2,439,270	90.99%
LSD	N6553	2,809,614	2,773,944	98.73%	2,537,693	91.48%
LSD	N7656	5,455,118	5,406,240	99.10%	4,934,249	91.27%
LSD	N7689	3,198,824	3,157,705	98.71%	2,903,225	91.94%
LSD	N7711	2,841,576	2,808,882	98.85%	2,598,070	92.49%
LSD	N8162	2,025,252	2,000,033	98.75%	1,832,833	91.64%
LSD	O0050	9,824,010	9,715,867	98.90%	8,718,051	89.73%
LSD	O2191	5,332,356	5,288,055	99.17%	4,911,915	92.89%

The individuals labeled in red were excluded from this study.

Table S2. Summary of raw read counts, quality control statistics, and genome mapping percentages for the nine whole-genome resequencing individuals.

Strain	Sample ID	Number of reads	Number of reads passing QC	QC percentage	Number of mapped reads	Mapping percentage
FD8X	P1175	126,329,362	109,169,275	86.42%	101,821,890	93.27%
FD8X	P1176	174,306,970	155,803,144	89.38%	143,416,852	92.05%
FD8X	O8537	156,899,682	139,493,006	88.91%	130,126,597	93.29%
FD8X	O8539	166,181,582	147,175,245	88.56%	137,382,425	93.35%
FD8X	P1172	131,783,274	115,138,533	87.37%	107,249,903	93.15%
FD8X	P1174	118,865,040	106,249,179	89.39%	99,433,749	93.59%
LSD	P1171	167,162,764	148,164,209	88.63%	140,168,328	94.60%
LSD	P1168	126,659,892	112,194,449	88.58%	106,082,598	94.55%
LSD	P1169	129,673,220	113,255,096	87.34%	107,184,413	94.64%

Table S3. Inbreeding coefficients calculated based on pedigree information for the 70 ddRAD-seq individuals.

Strain	Sample ID	Inbreeding coefficient	Strain	Sample ID	Inbreeding coefficient
AH11L	M9845	0.525	FD2M1	N9788	0.976
AH11L	N6251	0.4293	FD2M1	N9828	0.976
AH11L	N6901	0.7713	FD2M1	O0317	0.9843
AH11L	N7773	0.775	FD2M1	O0713	0.9818
AH11L	N7955	0.7679	FD2M1	O0838	0.9835
AH11L	N9649	0.7648	FD2M4	N6790	0.9297
AH11L	O0199	0.7696	FD2M4	N6806	0.923
AH11L	O0496	0.7686	FD2M4	N7364	0.9317
AH11L	O0824	0.77	FD2M4	N7367	0.9317
AH11L	O0832	0.77	FD2M4	N9017	0.923
ATHHN	N7056	0.7901	FD2M4	N9488	0.9307
ATHHN	N7101	0.7939	FD8X	N9040	0.2148
ATHHN	N7276	0.7975	FD8X	O1571	0.2255
ATHHN	N7464	0.7913	FD8X	O5150	0.2211
ATHHN	N7476	0.8183	FD8X	O6210	0.2264
ATHHN	N8487	0.7885	FD8X	O6669	0.2194
ATHHN	N8663	0.7885	FD8X	O6670	0.2194
ATHHN	O0936	0.7935	LL1	A0007	0
ATHHN	O0957	0.7935	LL1	A0027	0
ATHHN	O1303	0.7983	LL1	A0028	0
ATHL	N3283	0.936	LL1	A0050	0.0938
ATHL	N3836	0.9354	LL1	A0051	0
ATHL	N3838	0.9354	LL1	A0063	0
ATHL	N4852	0.9354	LL1	A0064	0
ATHL	N6224	0.9377	LL1	A0070	0
ATHL	N6282	0.9375	LSD	N6107	0.9816
ATHL	N6536	0.9353	LSD	N6111	0.9816
ATHL	N7036	0.9369	LSD	N6551	0.9816
ATHL	N8339	0.9414	LSD	N6553	0.9816
ATHL	N8500	0.9382	LSD	N7656	0.9816
FD2M1	N5168	0.9899	LSD	N7689	0.9816
FD2M1	N7405	0.9796	LSD	N7711	0.9816
FD2M1	N7616	0.9813	LSD	N8162	0.9816
FD2M1	N8389	0.9918	LSD	O0050	0.9851
FD2M1	N9534	0.9811	LSD	O2191	0.9879

Table S4. Population genetics parameters in the eight *Monodelphis domestica* strains.

Strain	% of heterozygous SNPs		Nucleotide diversity (π)		Fst	
	Autosomes mean (SD)	chrX mean (SD)	Autosomes mean (SD)	chrX mean (SD)	Autosomes mean (SD)	chrX mean (SD)
AH11L	0.000565 (0.000244)	0.000100 (0.000047)	0.001353 (0.004028)	0.001494 (0.008181)	0.098979 (0.189584)	0.094261 (0.210063)
ATHHN	0.000482 (0.000229)	0.000105 (0.000056)	0.001271 (0.003925)	0.001471 (0.008075)	0.103362 (0.190606)	0.091912 (0.208802)
ATHL	0.000420 (0.000298)	0.000025 (0.000005)	0.001117 (0.003685)	0.000881 (0.006791)	0.110805 (0.195390)	0.104664 (0.229060)
FD2M1	0.000365 (0.000207)	0.000031 (0.000008)	0.001504 (0.004151)	0.00305 (0.010123)	0.108621 (0.190581)	0.093446 (0.212486)
FD2M4	0.000283 (0.000167)	0.000041 (0.000013)	0.001278 (0.003821)	0.002678 (0.009867)	0.116350 (0.205725)	0.098770 (0.226274)
FD8X	0.000454 (0.000217)	0.000042 (0.000011)	0.002743 (0.006496)	0.002368 (0.009926)	0.095253 (0.167677)	0.079091 (0.182522)
LL1	0.000660 (0.000262)	0.000125 (0.000063)	0.002285 (0.005654)	0.004134 (0.012717)	0.073355 (0.129685)	0.064861 (0.144957)
LSD	0.000148 (0.000097)	0.000048 (0.000022)	0.001110 (0.003404)	0.001879 (0.008784)	0.115052 (0.195304)	0.087883 (0.194416)
