# Supplementary material for: Genetic and genomic architecture in eight strains of the laboratory opossum Monodelphis domestica
Source: G3 (Bethesda). 2021 Nov 9;12(1):jkab389. doi: 10.1093/g3journal/jkab389 (PMC8728031; doi:10.1093/g3journal/jkab389)
Supplement: jkab389_Supplementary_Figures-Tables-Captions [file jkab389_supplementary_figures-tables-captions.docx]

# Supplemental Materials

# Supplemental Figures

**Figure S1.** Histograms of proportions of homozygous/ heterozygous SNPs for eight autosomes of 70 individuals in eight opossum strains.

**Figure S2.** Barplot of numbers of heterozygous SNPs in female (left) and male (right) lab opossum samples.

**Figure S3.** Window-based nucleotide diversity plots along autosomes and the X chromosome for eight laboratory opossum strains (window size = 20kb).

**Figure S4.** Window-based Fst plots along autosomes and the X chromosome for eight laboratory opossum strains (window size = 20kb).

# Supplemental Tables

**Table S1.** Summary of raw read counts, quality control statistics, and genome mapping percentages for ddRAD-seq individuals.

**Table S2.** Summary of raw read counts, quality control statistics, and genome mapping percentages for the nine whole-genome resequencing individuals.

**Table S3.** Inbreeding coefficients calculated based on pedigree information for ddRAD-seq individuals.

**Table S4.** Population genetic parameters for the eight *Monodelphis domestica* strains.

# Supplemental Data

**Data S1.** *Monodelphis domestica* SNP ID, position, and genotype in ddRAD-seq and whole-genome resequencing individuals.

**Data S2.** SNP genotypes in RNA-seq data and ddRAD-seq data for 2,151 overlapped SNP positions.
